# Comparative Assessment of the Toxicity of Brominated and Halogen-Free Flame Retardants to Zebrafish in Terms of Tail Coiling Activity, Biomarkers, and Locomotor Activity

**DOI:** 10.3390/toxics11090732

**Published:** 2023-08-25

**Authors:** Taisa Carla Rizzi Rialto, Renan Vieira Marino, Flavia Renata Abe, Daniel Junqueira Dorta, Danielle Palma Oliveira

**Affiliations:** 1 Department of Clinical, Toxicological and Food Sciences, School of Pharmaceutical Sciences of Ribeirão Preto, University of São Paulo, Ribeirão Preto 14040-903, SP, Brazil; taisarialto@usp.br (T.C.R.R.); frabe@alumni.usp.br (F.R.A.); 2 Department of Chemistry, Faculty of Philosophy Science and Letters at Ribeirão Preto, University of São Paulo, Ribeirão Preto 14040-901, SP, Brazil; djdorta@ffclrp.usp.br; 3 National Institute for Alternative Technologies of Detection, Toxicological Evaluation and Removal of Micropollutants and Radioactives (INCT-DATREM), Araraquara 14800-900, SP, Brazil

**Keywords:** BDE-47, ALPI, *Danio rerio*, motor behavior, acetylcholinesterase activity, oxidative stress

## Abstract

BDE-47, a flame retardant that is frequently detected in environmental compartments and human tissues, has been associated with various toxic effects. In turn, information about the effects of aluminum diethyl-phosphinate (ALPI), a halogen-free flame retardant from a newer generation, is limited. This study aims to assess and compare the toxicity of BDE-47 and ALPI to zebrafish by analyzing the tail coiling, locomotor, acetylcholinesterase activities, and oxidative stress biomarkers. At 3000 µg/L BDE-47, the coiling frequency increased at 26–27 h post-fertilization (hpf), but the burst activity (%) and mean burst duration (s) did not change significantly. Here, we considered that the increased coiling frequency is a slight neurotoxic effect because locomotor activity was impaired at 144 hpf and 300 µg/L BDE-47. Moreover, we hypothesized that oxidative stress could be involved in the BDE-47 toxicity mechanisms. In contrast, only at 30,000 µg/L did ALPI increase the catalase activity, while the motor behavior during different developmental stages remained unaffected. On the basis of these findings, BDE-47 is more toxic than ALPI.

## 1. Introduction

For decades, polybrominated diphenyl ethers (PBDEs) have been extensively used as additive brominated flame retardants in commercial products, such as electronics, appliances, textiles, and household furnishing [1]. Because PBDEs do not chemically bind to these products, they can leach into the environment [2]. PBDEs often bind to particles in the atmosphere and accumulate in water, sediments, and soil and in human and animal fatty tissues, posing a great threat to the environment and human health [3]. Previous studies reported that concentrations of pg/L to ng/L PBDEs have been found in water samples, while pg/g to ng/g dw have been found in soil and sediments samples, and pg/m^3^ to ng/m^3^ in air [3,4,5,6,7,8,9,10].

BDE-47 is one of the most toxic congeners. In mammals, it has been reported to induce reproductive toxicity in mice [11] and to disturb the neurotransmitter system and the phosphorylation and mitochondrial processes in the mouse brain, inducing oxidative stress [12]. Overall, the authors’ results revealed that exposure to BDE-47 may be a risk factor for developing Parkinson’s disease due to aberrant protein aggregation originating from disturbed phosphorylation [12]. In a mouse model of diet-induced obesity with exposure to BDE-47 for 15 weeks, lipid deposition increased in the liver (hepatic steatosis), and oxidative stress-induced hepatic inflammation and liver fibrosis [13].

Several studies about the BDE-47 toxic effects on zebrafish (*Danio rerio*) have been published. BDE-47 affects zebrafish embryonic-larval development by inducing pericardium and yolk sac edemas [4]. In addition, low BDE-47 concentrations impair vascular development in zebrafish early stages [14], induce hyperactivity in zebrafish embryos by functionally inhibiting dopaminergic neurons [15], and negatively impact ovary development, decreasing sex hormone levels, inducing oxidative damage, and altering hypothalamic pituitary-gonad axis-related genes in female zebrafish [16].

In this context, searching for alternatives that are less toxic to the environment and human health is necessary. Aluminum diethyl-phosphinate (ALPI) belongs to a new generation of halogen-free flame retardants and is mainly used in the engineering plastics segment, for example, in electrical and electronic equipment, connectors, switches, and encapsulated electronic components [17]. In mammals, sub-chronic and single oral exposure to ALPI has been associated with mild toxicity to the liver and reproductive system [18], but it does not induce morphological abnormalities during zebrafish embryonic development [4]. Furthermore, few studies are available regarding the environmental detection of ALPI. Among the limited studies addressing the environmental detection of ALPI, one study that evaluated samples collected around a manufacturing plant found concentrations varying between 10.8 and 34.0 μg/kg in soil and from 28.3 to 1795.1 μg/kg in sediment [19].

Zebrafish are a useful vertebrate model for studying the toxicity of hazardous chemicals to organism development and the reproductive, cardiovascular, neural, and ocular systems [20]. They are also a powerful model for investigating oxidative phenomena during development [21]. These organisms have rapid embryonic development—this stage lasts about 72 h post-fertilization (hpf)—which is essential when investigating the early warning effects of hazardous chemicals. For instance, the tail coiling assay evaluates spontaneous side-to-side contractions of the trunk in zebrafish embryos from 26.0 to 28.5 hpf. This rapid assay can be chosen to assess developmental neurotoxicity [22]. At the larval stage, the general zebrafish swimming phenotype can be evaluated to indicate depressive or upregulated-like behaviors associated with neural disorders [23]. Indeed, behavior is a sensitive and environmentally relevant endpoint for neurotoxicity research, and zebrafish have emerged as a key model for studying fish behavior [24]. Furthermore, behavior endpoints represent an integrated organism response in which biochemical and physiological processes are linked.

Therefore, this study aimed to carry out a comparative toxicity assessment of BDE-47 and ALPI; zebrafish early stages were employed as an organism model. We assessed the tail-coiling activity of very early embryos and the locomotor activity of larvae and addressed the motor behavior in different stages. We quantified the activity of the enzyme acetylcholinesterase (AChE), an important marker of cholinergic synapses and neuromuscular junctions [25,26], as well. Given the potential of flame retardants to induce oxidative damage, we also assessed biochemical biomarkers involved in oxidative stress in zebrafish larvae.

## 2. Materials and Methods

### 2.1. Chemicals

The brominated congener BDE-47 (2,2′,4,4′ -tetrabromodiphenyl ether, CAS n° 5436-43-1, MW 485.79, 99.9% purity) was purchased from AccuStandard (New Haven, CT, USA). ALPI (aluminum diethyl-phosphinate, CAS n° 225789–38–8, MW 390.27) was donated by Clariant (trade name of Exolit^®^ OP 1230) (Clariant, Germany). BDE-47 stock solutions were prepared in 100% dimethyl sulfoxide (DMSO) and diluted in embryo medium (2 mM CaCl_2_, 0.5 mM MgSO4, 0.75 mM NaHCO_3_, and 0.07 mM KCl) to the tested concentration (0.01% or 0.1% DMSO, *v*/*v*). ALPI was prepared in embryo medium and left on a shaker at 50–80 rpm overnight for complete dissolution, which was followed by dilution in embryo medium to prepare the tested concentrations. Embryo medium and 0.01% or 0.1% DMSO in embryo medium (*v*/*v*) were used as negative controls.

### 2.2. Test Organism

Adult wild-type zebrafish (*Danio rerio*) were kept at the Environmental Toxicology Animal Facility of the School of Pharmaceutical Sciences of Ribeirão Preto, University of São Paulo (Ribeirão Preto, Brazil). They were maintained in a ZebTEC system (Tecniplast, Italy) under standard conditions (temperature = 26 ± 1 °C, pH = 7.5 ± 0.5, dissolved oxygen at 95% saturation, conductivity = 750 ± 50 μs/cm, 14:10 h light/dark photoperiod) and fed with TetraMin^®^ Tropical Granules. Male and female fish (2:1 ratio) were placed into a 1 L breeding tank for egg acquisition. At the beginning of the light photoperiod, they were left to spawn for approximately 90 min. The eggs were collected and rinsed in embryo medium. Fertilized eggs were randomly selected under a stereomicroscope for the subsequent assays. The protocol was approved by the Ethics Committee on Animal Use of the University of São Paulo (CEUA-USP), protocols 19.1.845.60.4 and 19.1.761.60.5.

### 2.3. Tail Coiling Assay

Treatments consisted of the negative controls (embryo medium or 0.1% DMSO in embryo medium, *v*/*v*) and three concentrations of each flame retardant—3, 300, or 3000 µg/L—prepared as described in Section 2.1. Sub-lethal concentrations were selected from our previous studies [4] and considering the final solvent concentration that did not impact embryo activity. For each chemical compound, embryos up to the late blastula period (sphere stage, 4 hpf) were transferred to six-well plates (20 embryos per well) filled with 10 mL of flame retardant test solution or negative control. Immediately, plates were incubated at 27.5 ± 0.5 °C under 13:11 h light/dark photoperiod. The test was performed at 26 hpf in independent triplicates at the same time interval, in accordance with de Oliveira, Brigante, and Oliveira [22]. Approximately 20 non-dechorionated embryos from each treatment were transferred to single concave glass slides (75 mm × 25 mm) and acclimated for 2 min to minimize disturbance during embryo transfer. Room light and temperature were kept constant (25 °C). The tail coiling activity was recorded for 3 min by using a camera (AxioCam IC 5, Zeiss, Germany) coupled to a stereomicroscope (Stemi 508, Carl Zeiss, Germany). The recording order of the treatments was different for each replicate and lasted 1 h on average. During video monitoring, the embryos did not receive any sensory stimulation. Movies were obtained at 10 frames per second (fps) and processed in the Audio/Video Interleaved (AVI) (Motion-JPEG compression) format. Raw data were extracted and analyzed with the software DanioScope (Noldus, version: 1.1, Wageningen, The Netherlands). The coiling activity is presented as the percentage of time the embryo was moving (“burst activity”), the sum of all movement durations (“mean burst duration”), and the number of embryo movements per minute (“burst count per minute”).

### 2.4. Locomotor Activity

The locomotor activity was evaluated according to Henriques et al. [27] and Abe et al. [28], with slight adaptations. Sub-lethal concentrations were selected from our previous studies [4] using the final solvent concentration as low as possible (0.01%). Treatments consisted of the negative controls (embryo medium or 0.01% DMSO in embryo medium, *v*/*v*), five BDE-47 concentrations (0.03, 0.3, 3, 30, or 300 µg/L), and five ALPI concentrations (3, 30, 300, 3000, or 30,000 µg/L), prepared as described in Section 2.1. For each chemical compound, viable eggs, up to the late blastula period, were carefully placed in six-well plates at a ratio of 1 egg per 1 mL of flame-retardant test solution or negative control. The plates were wrapped in parafilm and incubated at 27.5 ± 0.5 °C under 13:11 h light/dark cycle photoperiod. At 144 hpf, the larvae were individually transferred to 96-well plates (n = approximately 24 larvae/treatment/replicate) containing 300 μL of each tested flame-retardant concentration. To minimize disturbance during transfer of the larvae, they were acclimated in the 96-well plates for 60 min before locomotion was tracked. Locomotion tracking was recorded between 14:20 and 16:20 h [29] in independent triplicates; the software video tracking systems ZebraBox and ZebraLab (ViewPoint Life Science, Lyon, France) were used. For the first 10 min, tracking (acclimation period) comprised 5 min dark cycle + 5 min light cycle. After that, four consecutive 10 min cycles were recorded; dark and light were interspersed. The detection limit of 30 frames per second was used to detect the larvae. Movements slower than 2 mm/s were standardized as “inactive” to avoid system noise tracking, and movements faster than 20 mm/s were set as bursting behavior. The integration period (the intervals among the output data) took place every 60 s. The output data were total swimming distance (mm) and total swimming time (s). Immediately after video tracking, the larvae were analyzed under a stereomicroscope (Stemi 508, Carl Zeiss, Germany) to verify whether the swim bladder was inflated, given that an uninflated swim bladder can impair swimming [30].

### 2.5. Biochemical Analyses

Sub-lethal concentrations were also selected from our previous studies [4] using the final solvent concentration as low as possible (0.01%). Treatments consisted of the negative controls (embryo medium or 0.01% DMSO in embryo medium, *v*/*v*), five BDE-47 concentrations (0.03, 0.3, 3, 30, or 300 µg/L), and five ALPI concentrations (3, 30, 300, 3000, or 30,000 µg/L), prepared as described in Section 2.1. For each chemical compound, viable eggs, up to the late blastula period, were carefully placed in six-well plates (15 eggs per well) filled with 10 mL of the flame-retardant test solution or negative control. The plates were wrapped in parafilm and incubated at 27.5 ± 0.5 °C under 13:11 h light/dark photoperiod. After 96 hpf, 6 clusters of 15 hatched larvae per treatment and controls were collected in 2 mL ice-covered microtubes, totaling approximately 90 larvae for each tested concentration or negative control. The larvae were frozen by immersion in liquid nitrogen and immediately stored at −80 °C until analysis [31,32]. The samples were defrosted and homogenized on ice in 800 µL of ultrapure water; a sonic homogenizer was employed. Then, 500 μL of the homogenate was mixed with 500 μL of potassium phosphate buffered saline (0.2 M; pH 7.4) and subsequently centrifuged (10,000× *g*; 4 °C; 20 min) to isolate the post-mitochondrial supernatant (PMS). The supernatant fraction was distributed in new microtubes to determine AChE (250 μL), CAT (100 μL), GST (250 μL), and proteins (100 μL). Biochemical analyses of the samples were performed spectrophotometrically in quadruplicate or triplicate at 25 °C on a Thermo Scientific Multiskan Sky Microplate™ Reader (Waltham, MA, USA).

#### 2.5.1. Acetylcholinesterase (AChE)

The AChE activity was determined according to Ellman et al. [33] and adapted for microplates as described by Gravato et al. [34] and Guilhermino et al. [35] on the basis of the rate of thiocholine production from acetylthiocholine iodide hydrolysis. The activity was monitored at 414 nm for 5 min and expressed as nmol/min/mg of protein.

#### 2.5.2. Catalase (CAT)

The CAT activity was determined according to Claiborne [36] by following the decline in absorbance resulting from H_2_O_2_ consumption. The activity was monitored for 2 min at 240 nm and expressed as μmol/min/mg of protein.

#### 2.5.3. Glutathione S-Transferase (GST)

The GST activity was determined according to Habig, Pabst, and Jakoby [37] and adapted for microplates by Frasco and Guilhermino [38]; glutathione (GSH) conjugation to the substrate 1-chloro-2,4-dinitrobenzene (CDNB) was measured at 340 nm for 5 min. The enzymatic activity was expressed as nmol/min/mg of protein.

#### 2.5.4. Protein Quantification

Proteins were quantified as described by Bradford [39] and adapted for microplates by Guilhermino et al. [35]. Bovine γ-globulin was used as standard, and spectrophotometric measurement was performed at 600 nm.

### 2.6. Statistical Analysis

The experimental data were statistically analyzed by using the software GraphPad Prism 8.4.3 (GraphPad Software, San Diego, CA, USA). Data from the tail coiling assay and light cycles (Light I + Light II) of locomotor activity were analyzed by Kruskal–Wallis (ANOVA-on-ranks), and mean differences were verified with Dunn’s post-hoc test. Data from the biochemical analyses and dark cycles (Dark I + Dark II) of locomotor activity were analyzed by one-way ANOVA followed by Dunnett’s post-hoc test to verify significant differences between treatments and negative controls. Significance was set at *p* < 0.05 (*). Data are presented as scatter plot with bars depicted by mean ± standard deviation (SD).

## 3. Results

The tested concentrations did not cause malformations nor mortality in exposed embryos in all assays performed, as already described by Abe et al. [4]. The results for each assay are presented below.

### 3.1. Tail Coiling Assay

**Figure 1:**
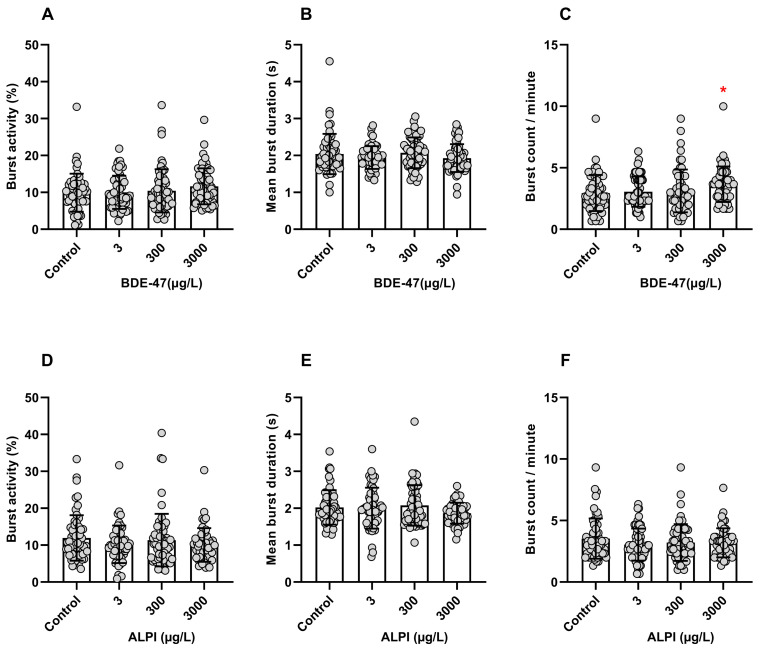
Coiling activity of zebrafish (*Danio rerio*) embryos aged 26 h post-fertilization (hpf) following exposure to flame retardants. Results are expressed as scatter plot with bars depicted by mean ± standard deviation. (**A**–**C**) BDE-47. n = 56–58. * Significant differences from the control (0.1% DMSO, *v*/*v*) for *p* < 0.05 (Kruskal–Wallis followed by Dunn’s post-hoc test). (**D**–**F**) ALPI. n = 52–60. No significant differences were found in relation to the control (embryo medium) for *p* < 0.05 (Kruskal–Wallis followed by Dunn’s post-hoc test).

Zebrafish embryos exposed to BDE-47 did not show significantly different burst activity (%) (H = 5.066; *p* > 0.05) or mean burst duration (s) (H = 4.870; *p* > 0.05). In contrast, the burst count per minute increased significantly at the highest tested concentration of 3000 µg/L BDE-47 when compared to the control (0.1% DMSO, *v*/*v*) (H = 9.911; *p* < 0.05) (Figure 1A–C). Embryos exposed to ALPI did not present significant differences in any of the analyzed parameters, namely burst activity (%) (H = 2.503; *p* > 0.05), mean burst duration (s) (H = 3.335; *p* > 0.05), and burst count per minute (H = 1.997; *p* > 0.05), when compared to the control (embryo medium) (Figure 1D–F).

### 3.2. Locomotor Activity

**Figure 2:**
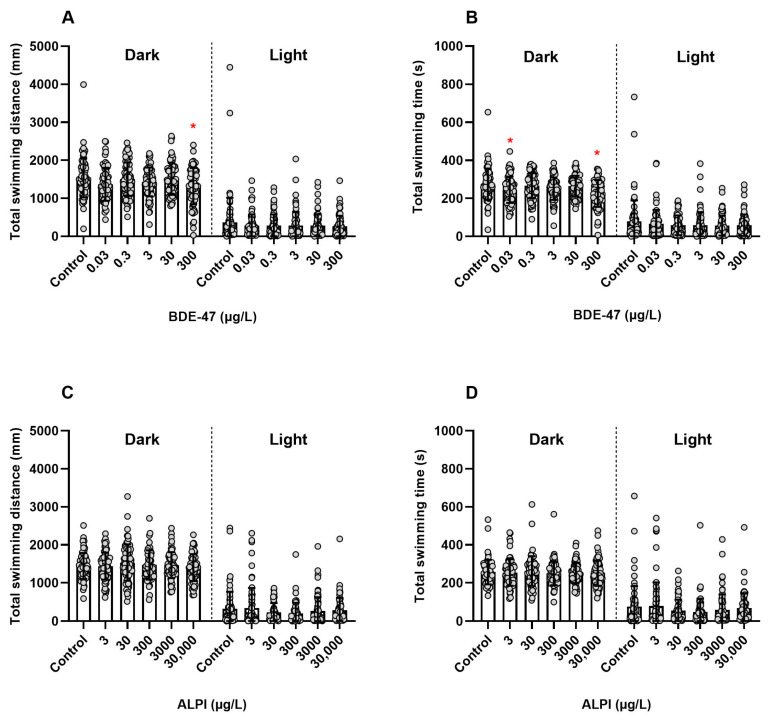
Locomotor activity of zebrafish (*Danio rerio*) larvae exposed to flame retardants along 144 h post-fertilization (hpf). Results are presented as scatter plot with bars depicted by mean ± standard deviation. (**A**,**B**) BDE-47. n = 72. * Significantly different from the control (0.01% DMSO, *v*/*v*) for *p* < 0.05 (one-way ANOVA followed by Dunnett’s post-hoc test for dark cycle and Kruskal–Wallis followed by Dunn’s post-hoc test for light cycle). (**C**,**D**) ALPI. n = 68–72. No significant differences were found in relation to the control (embryo medium) for *p* < 0.05 (one-way ANOVA followed by Dunnett’s post-hoc test for dark cycle and Kruskal–Wallis followed by Dunn’s post-hoc test for light cycle).

BDE-47 significantly decreased the total swimming distance (mm) (Dark I + Dark II: (F _(5, 426)_ = 2.838, *p* < 0.05)) and total swimming time (s) (Dark I + Dark II: (F _(5, 426)_ = 5.019, *p* < 0.05)) of larvae exposed to 300 µg/L BDE-47 when compared to the control (0.01% DMSO, *v*/*v*) (Figure 2A,B). Furthermore, the total swimming time (s) decreased significantly at 0.03 µg/L BDE-47 (Figure 2B). On the other hand, zebrafish larvae exposed to ALPI did not show significantly different total swimming distance (mm) or total swimming time (s) (Dark I + Dark II: F _(5, 418)_ = 0.9575, *p* > 0.05; Light I + Light II: H = 7.499, *p* > 0.05; and Dark I + Dark II: F _(5, 418)_ = 0.3319, *p* > 0.05; Light I + Light II: H = 9.088, *p* > 0.05; respectively) when compared to the control (embryo medium), as presented in Figure 2C,D.

### 3.3. Biochemical Analyses

**Figure 3:**
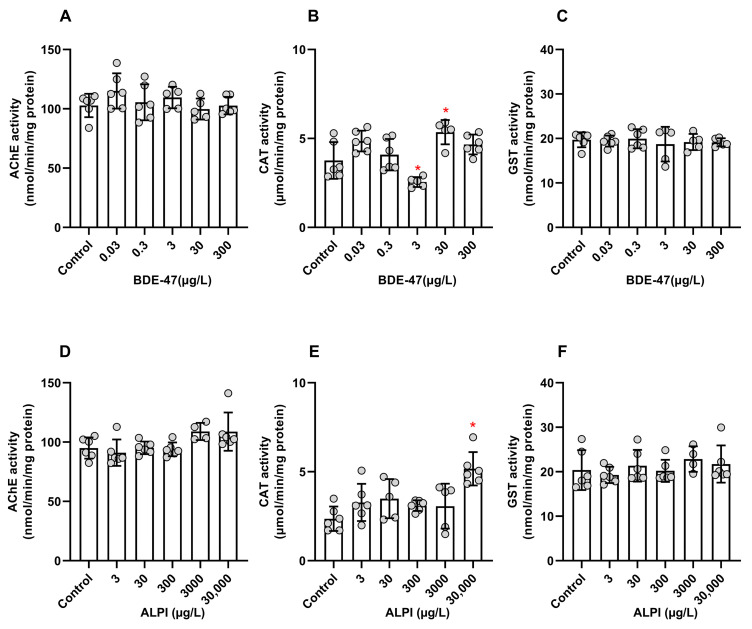
Activities of acetylcholinesterase (AChE), catalase (CAT), and glutathione S-transferase (GST) of zebrafish (*Danio rerio*) larvae exposed to flame retardants along 96 h post-fertilization (hpf). Results are expressed as scatter plot with bars depicted by mean ± standard deviation. (**A**–**C**) BDE-47. n = 5–6 clusters carrying 15 larvae each. * Significantly different from the control (0.01% DMSO, *v*/*v*) for *p* < 0.05 (one-way ANOVA followed by Dunnett’s post-hoc test). (**D**–**F**) ALPI. n = 4–6 clusters carrying 15 larvae each. * Significantly different from the control (embryo medium) for *p* < 0.05 (one-way ANOVA followed by Dunnett’s post-hoc test).

BDE-47 and ALPI significantly altered the CAT activity. For the other enzymatic activities analyzed here, the effects of the flame retardants did not differ significantly. For BDE-47, the AChE activity was not significantly altered (F _(5, 28)_ = 1.330; *p* > 0.05); the CAT activity decreased for 3 and increased for 30 µg/L (F _(5, 28)_ = 9.656; *p* < 0.05); and the GST activity remained unchanged (F _(5, 27)_ = 0.2295; *p* > 0.05) when compared to the control (0.01% DMSO, *v*/*v*) (Figure 3A–C). For ALPI, the AChE activity did not change (F _(5, 28)_ = 3.371); the CAT activity increased at the highest tested concentration of 30,000 µg/L (F _(5, 28)_ = 6.218; *p* < 0.05); and there were no significant effects on the GST activity (F _(5, 28)_ = 0.7026; *p* > 0.05) (Figure 3D–F).

## 4. Discussion

In this study, we comparatively assessed the neurotoxicity of BDE-47, a flame retardant known to be toxic, and a new alternative flame retardant, ALPI, by evaluating the tail coiling and locomotor activities of zebrafish embryos/larvae. Moreover, we assessed the activity of the enzyme AChE and the oxidative stress status of the exposed organisms by determining biochemical biomarkers. For all assays performed, and according to Abe et al. [4], the tested concentrations did not cause malformations or mortality in exposed embryos. BDE-47 slightly increased the coiling frequency of early embryos and impaired the locomotor activity of larvae without affecting the AChE activity. BDE-47 also altered the CAT activity of larvae. On the other hand, ALPI only increased the CAT activity significantly at the highest tested concentration.

The tail coiling assay allows developmental toxicity to be assessed at early zebrafish stages and addresses potential neurotoxic effects. In our study, burst count/minute (frequency) increased significantly in early embryos exposed to the highest tested concentration of 3000 μg/L BDE-47, showing that BDE-47 has neurotoxic potential. However, it was not clear whether it caused hyperactivity because the other parameters, burst activity (%) and mean burst duration (s), were not significantly altered. The increased coiling frequency agreed with the literature. For example, Usenko et al. [40] reported that BDE-28 and BDE-47 (tri and tetra BDE, respectively) increased spontaneous movement/coiling (flexes/min) at 24 hpf, showing that BDE-28 (≈20 mg/L) and BDE-47 (≈30 mg/L) more than doubled the rate of spontaneous movement of the embryos. In addition, Zhuang et al. [41] linked a significant increase in coiling frequency with the neurotoxic effects of BDE-47, which, at 1.250 mg/L, increased the zebrafish coiling frequency at 24 hpf due to increased serotonin and dopamine contents. BDE-47 also decreased the nestin levels of embryos significantly. Nestin is a protein implicated in the survival and renewal of neuro stem/progenitor cells, and decreased levels of this protein indicate that neurogenesis was disturbed. Indeed, Chen et al. [42] and Zheng et al. [2] showed that BDE-47 significantly increased the spontaneous coiling activity frequency and caused a deficit in the zebrafish swimming behavior and that these changes were linked to altered neuronal connectivity patterns, such as inhibited axonal growth and early neurogenesis development. Furthermore, Wang et al. [43] observed that the BDE-47 hydroxyl-metabolic product, 6-OH-BDE-47, increased the coiling frequency in zebrafish embryos, and this behavioral change has been linked to induced apoptosis in the brain. On the other hand, ALPI did not affect any parameter analyzed during the tail coiling assay, and so far, there is no neurobehavioral research available in zebrafish embryos.

Considering that higher tail coiling activity frequency could be associated with early impaired neurological functions in zebrafish, we verified whether the locomotion activity could be affected in the post-embryonic stage. Locomotor activity levels are usually used as a marker of behavioral responses, which can be analyzed by high-throughput platforms with the aid of video cameras (e.g., Zebrabox™) [44]. Here, after short alternations of dark and light phases during the assay, a robust locomotion pattern was triggered in larvae from the control group. During short dark periods, characteristic hyperactivity occurs and is defined as “dark photokinesis”, an undirected movement that allows larvae to return to illuminated areas to avoid darkness [23,24,45].

Regarding the locomotor activity of larvae exposed to BDE-47, the highest tested concentration of 300 µg/L BDE-47 significantly decreased the total swimming distance (mm) and total swimming time (s) during dark cycles (Dark I + Dark II), suggesting that BDE-47 negatively affected the motor activity of larvae. These results agreed with literature studies demonstrating that BDE-47 caused a deficit in the locomotor behavior of zebrafish larvae [46,47]. On the other hand, ALPI did not change the locomotor activity of larvae at any evaluated parameter. To our knowledge, there is no data available about ALPI effects on the locomotor behavior of zebrafish larvae. It is noteworthy that exposure occurred at ALPI concentrations up to 30,000 µg/L, 100-fold higher than the BDE-47 concentration at which we observed impaired locomotor activity.

Behavior endpoints are useful tools to assess external neurotoxicity expression caused by environmental pollutants and represent an integrated, whole-organism response that links biochemical and physiological processes [48,49]. Impaired locomotor activity may be related to altered neurotransmission systems and modified activity of several biomarkers. Wang et al. [50] observed that the flame retardant DE-71 impaired the cholinergic system and locomotor activity of zebrafish larvae by dysregulating calcium homeostasis. Moreover, zebrafish locomotion is regulated by the dopaminergic system [51,52], which is known to be disturbed by BDE-47 [15,41]. Indeed, harmed locomotor behavior, given by limited moving capability, can affect the survival of larvae and is a potential ecological threat [46].

On the basis of the results of the motor behavior of zebrafish embryos/larvae, we performed biochemical analyses to determine the cholinergic activity of larvae to investigate whether the mechanisms involved in flame retardant toxicity could disturb the neurotransmitter system. We investigated the enzyme AChE, which plays a crucial role in proper nervous system functioning by cleaving the neurotransmitter acetylcholine into acetic acid and choline [26]. The AChE activity is widely used as a biomarker of neurotoxicity. When this enzyme is inhibited, it can no longer hydrolyze acetylcholine, resulting in extended action of this neurotransmitter within the synaptic cleft. This prolonged activity disrupts the nervous system’s normal functioning and increases skeletal muscle contractions. Such disruptions can result in behavioral impairment and even death [25].

In the present research, we did not find that any of the tested flame retardants significantly affected the AChE activity of exposed larvae. In contrast, studies related to several PBDEs have indicated that they may alter the nervous system through the acetylcholine system. The PBDE mixture DE-71 significantly inhibited the AChE activity in zebrafish offspring after parental exposure [53]; the mixture also decreased acetylcholine contents in zebrafish larvae [50]. Exposure to BDE-209 and its mixtures with BDE-47 and BDE-99 also significantly inhibited the AChE activity in goldfish (*Carassius auratus*) [54]; BDE-209 alone decreased the AChE activity of the fish *Labeo rohita* after short-term exposure (24–48 h). Nevertheless, after this period, the AChE activity began to increase slightly, indicating that exposure to PBDE had a reversible effect on the AChE activity [26]. Additionally, long-term exposure (30 days) of mussels (*Mytilus galloprovincialis*) to 2–15 µg/L BDE-47 significantly inhibited the AChE activity in a reversible manner, with pre-exposure levels being recovered after depuration for 10 days [55]. The absence of BDE-47 effects on the AChE activity in our study could be related to the very low concentrations used herein, which might have been insufficient to alter the AchE activity significantly. Concerning ALPI, despite the absence of effects, the US EPA [56] reported moderate neurotoxicity on the basis of the analogy with aluminum hydroxide and professional judgment made by using rats as an experimental model. Therefore, further studies may be required to elucidate the neurotoxicity of flame retardants to the cholinergic system.

Considering that flame retardants can potentially induce oxidative stress, we performed a biochemical analysis to investigate the defense system status of the exposed organisms. The antioxidant defense system is composed of enzymatic and non-enzymatic antioxidants that act by eliminating reactive oxygen species (ROS) and protecting the organism from oxidative damage. The first line of defense against oxidative stress involves the antioxidant enzymes superoxide dismutase (SOD), catalase (CAT), and glutathione peroxidase (GPx). SOD converts the oxygen-free radicals to hydrogen peroxide molecules, whereas CAT degrades hydrogen peroxide into water and molecular oxygen [57]. GST is another key enzyme involved in the metabolism of pollutants—it catalyzes the combination of glutathione (GSH) with electrophilic metabolites, facilitating their excretion [58]. Within the first hours of embryogenesis (as early as 4 hpf), zebrafish already present expressed GST enzymes, which are active and capable of catalyzing GSH conjugation with toxicants [59].

In the present study, BDE-47 and ALPI changed the CAT activity, but none of these flame retardants altered the GST activity significantly. Our results agreed with the results reported by Usenko et al. [60], who did not observe significant effects on the GST activity after BDE-47 zebrafish embryos (24 and 120 hpf) were exposed to BDE-47 (10 ppm). The results of transcriptional responses to oxidative stress indicated that PBDEs did not induce oxidative stress. According to these authors, these chemicals should not initiate ROS production due to their structural or chemical features. However, PBDE metabolism should result in hydroxylated products that might increase the potential for biological reactivity. Indeed, recent literature has shown that exposure to BDE-47 induced excessive ROS production in zebrafish embryos [61], increased the SOD and CAT activities in adult zebrafish liver [62], generated oxidative stress, and increased the defense system activity in adult zebrafish ovary, with significantly higher SOD activity and greater levels of malondialdehyde, one of the most significant membrane lipid peroxidation end products [16]. Moreover, Wang et al. [63] observed that exposure to low BDE-47 concentrations at 96 hpf caused the SOD, CAT, and GPx activities and malondialdehyde levels to be upregulated because ROS were overproduced in zebrafish larvae.

For BDE-47, the CAT activity displayed an inverted U-shaped dose response, which could be related to the overloaded defense system at the highest tested concentration of 300 µg/L. Indeed, at this concentration, the locomotor activity of exposed larvae decreased. Thus, although the larvae had elevated defense system activity, this activity may have been overwhelmed at high BDE-47 concentrations, leading the system to fail and other toxic effects to appear. As for ALPI, one of the few studies available about it showed that oxidative stress was generated in in vitro cell cultures: 140 μM ALPI increased ROS production in PC12 pheochromocytoma and rat B35 neuroblastoma cells [64]. Furthermore, although impaired locomotor activity indicates oxidative stress caused ALPI, we did not observe that here.

## 5. Conclusions

BDE-47 increases the coiling frequency in embryos without significantly altering the burst activity and mean burst duration. Although the results of this study point to a slight BDE-47 neurotoxic effect, we suggest that such an effect can be an early indicator of brominated flame retardant-induced neurotoxicity, given that increasing exposure to this compound causes locomotor hypoactivity in zebrafish larvae. Nevertheless, the AChE activity remains unchanged. Oxidative stress could underlie BDE-47 toxicity. For ALPI, on the other hand, there is only an indication of oxidative stress without motor behavior impairment in different developmental stages. Therefore, on the basis of our findings, the brominated flame retardant has higher toxicity than the halogen-free flame retardant. Finally, information regarding ALPI toxicity is lacking in all the assays performed in this study. According to this toxicity assessment in different stages of zebrafish development, and despite the need for further toxicity studies, this research contributes new information that can help regulatory agencies to select new flame retardants that are safer for the environment and humans.

## Data Availability

The data presented in this study are available on request from the corresponding author.
